# Supplementary material for: HOT or not: examining the basis of high-occupancy target regions
Source: Nucleic Acids Res. 2019 May 22;47(11):5735–45. doi: 10.1093/nar/gkz460 (PMC6582337; doi:10.1093/nar/gkz460)
Supplement: gkz460_Supplemental_Files [file gkz460_supplemental_files.zip › SupplementaryFiguresMaterials.pdf]

Figure S1

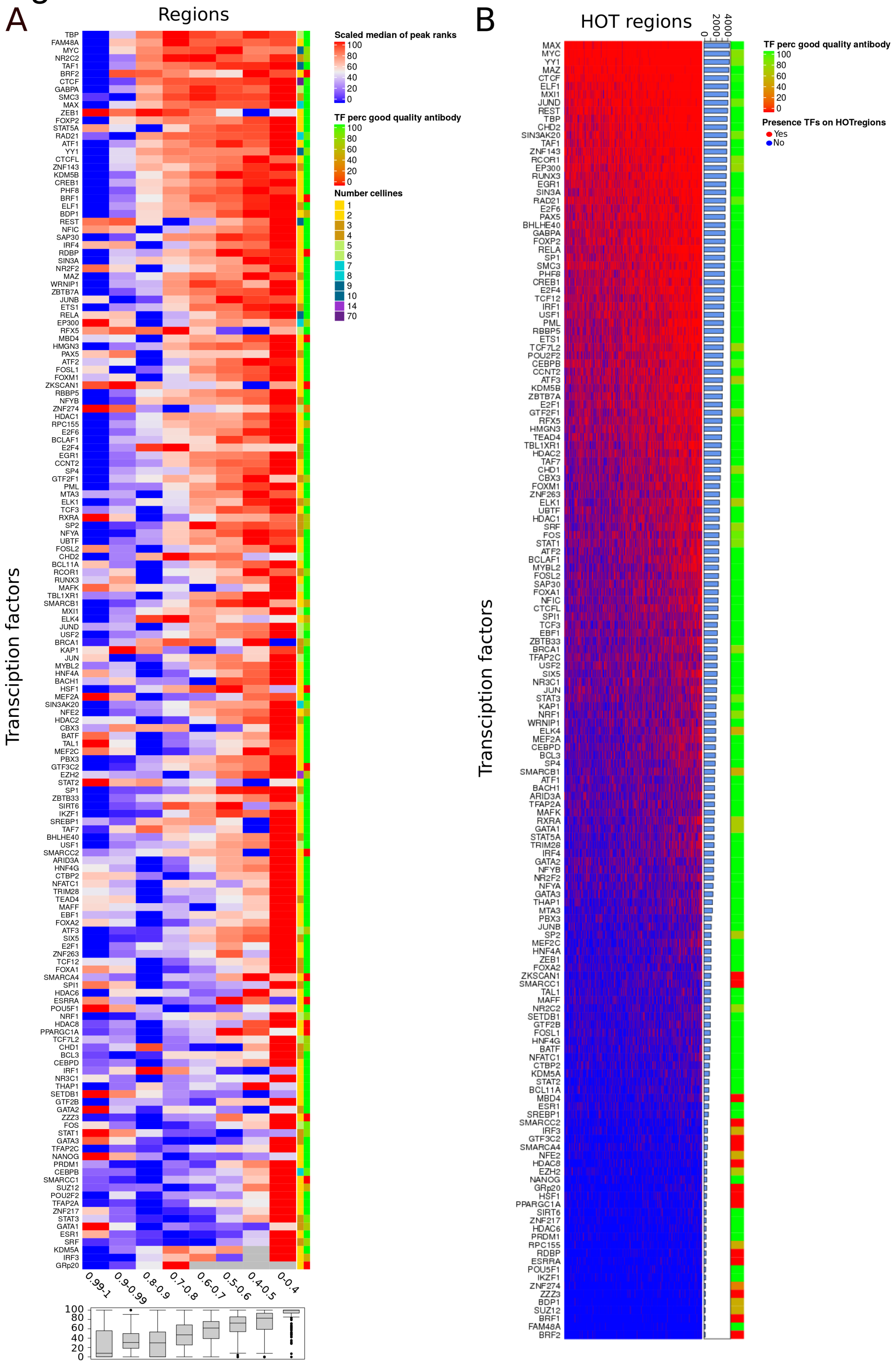
A
Regions
Transcription factors
Scaled median of peak ranks
TF perc good quality antibody
Number cellines
0.99-1
0.9-0.99
0.8-0.9
0.7-0.8
0.6-0.7
0.5-0.6
0.4-0.5
0-0.4
B
HOT regions
0
2000
4000
Transcription factors
TF perc good quality antibody
Presence TFs on HOTregions
Yes
No

Figure S1. A) HOT peaks are top rank TF peaks for majority of TFs. A heatmap shows median of ranks of TFs per HOT region (the highest rank is 1, the lowest 100). In comparison to less HOT regions, HOT region have the lowest median of ranks (see boxplots to the right of the heatmap). Annotation bars above the heatmap show percentage of good quality of antibodies (quality is per experiment is either good or caution) and number of cell lines per TF. **B)** Distribution of different TFs on HOT regions. Heatmap shows presence/absence of TFs on HOT regions. Hot regions are sorted from the most HOT regions on top to the less HOT regions in bottom. Annotation bar of quality of antibodies is as in S1A and the barplot indicates number of HOT regions present in each TF. The scatterplot to the right from the heatmap shows number of Tfs per HOT region.

Figure S2

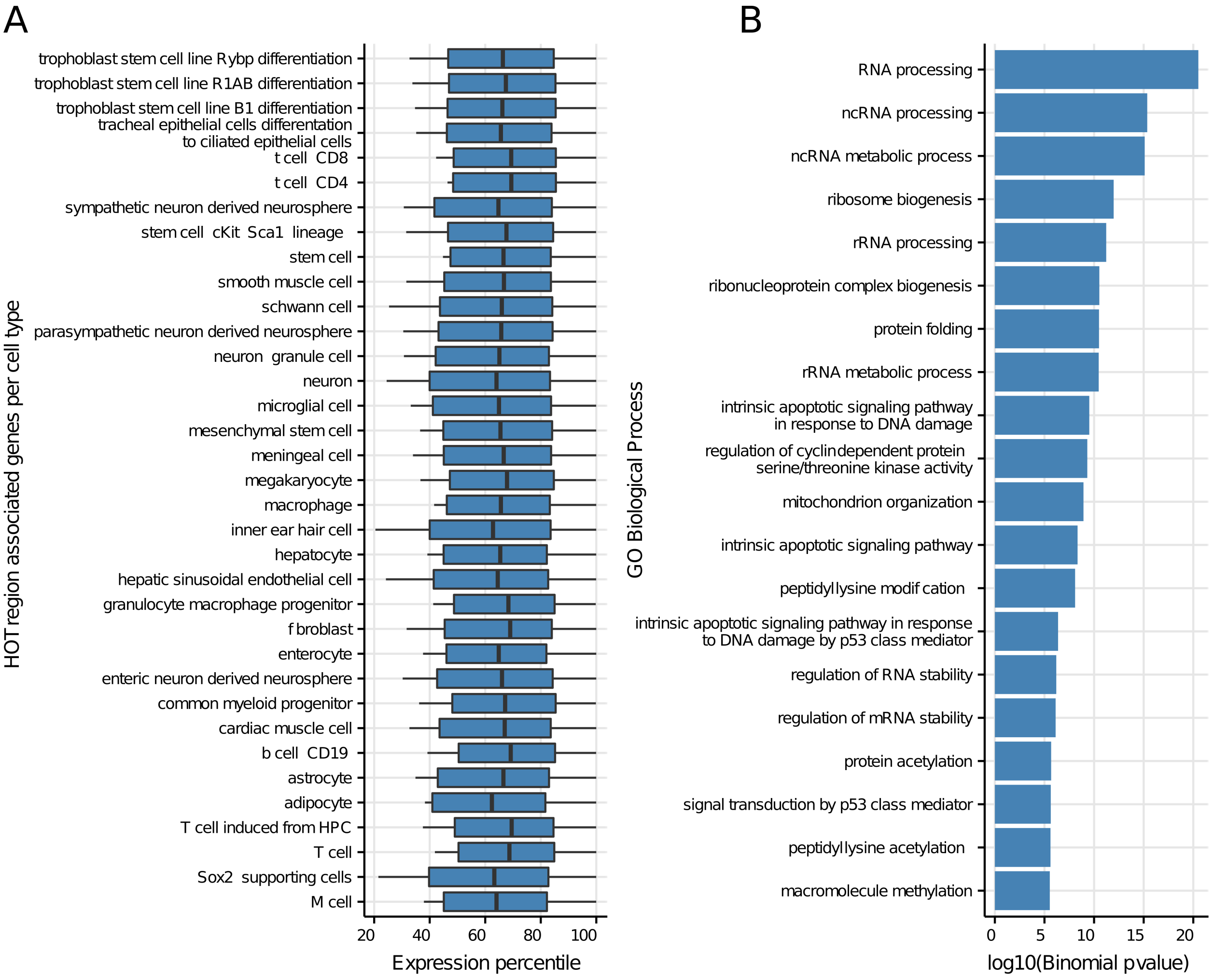

Figure S2. A) Expression profiles of genes associated with HOT regions across cell types in mouse (Expression Atlas EBI databases using fantom5 CAGE expression). The genes are stably expressed in all 35 cell types between 40th and 80th percentile. **B)** Functional enrichment analysis with Gene Ontology and and KEGG pathway on genes associated with murine HOT regions. HOT regions are significantly enriched for terms that relate to housekeeping functions.

Figure S3

A

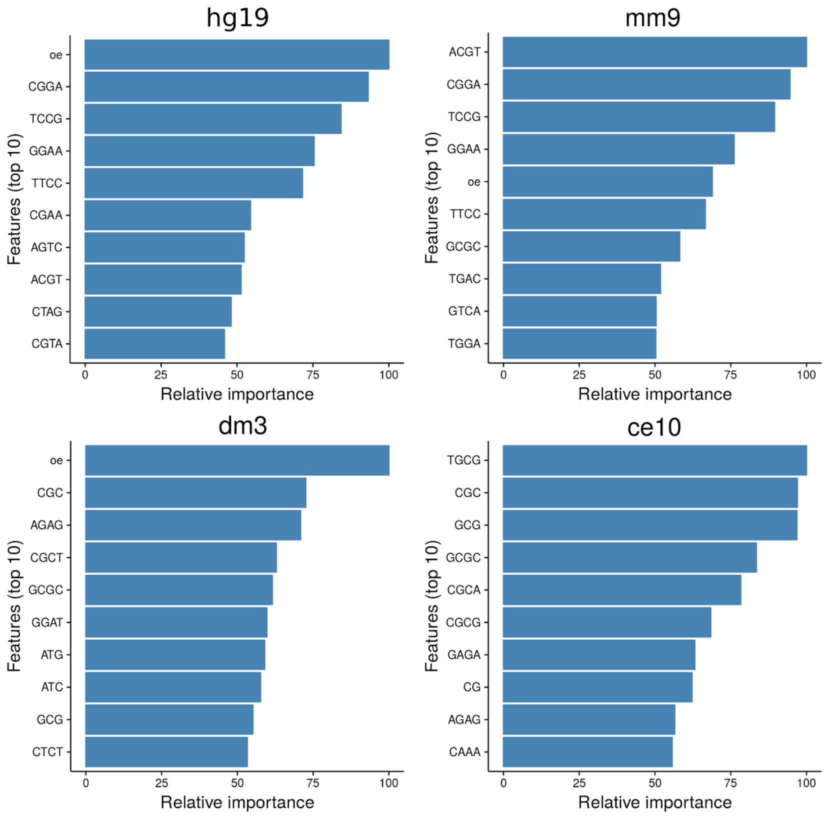

B

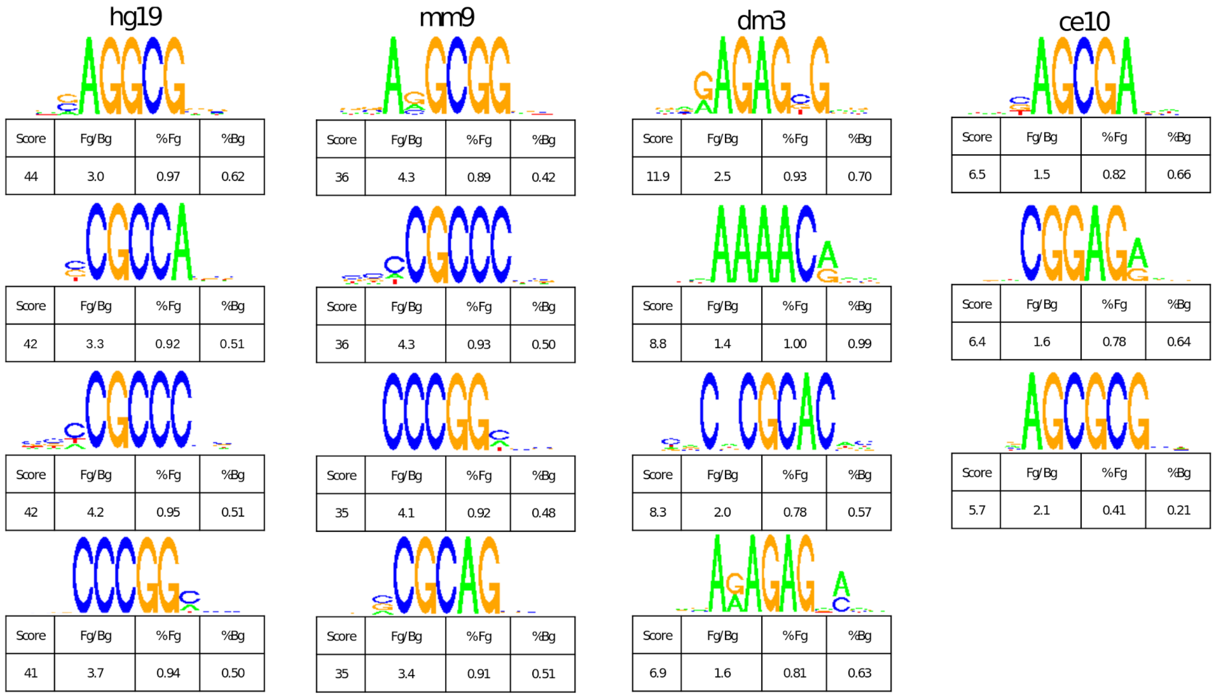

hg19

Score	Fg/Bg	%Fg	%Bg
44	3.0	0.97	0.62

Score	Fg/Bg	%Fg	%Bg
42	3.3	0.92	0.51

Score	Fg/Bg	%Fg	%Bg
42	4.2	0.95	0.51

Score	Fg/Bg	%Fg	%Bg
41	3.7	0.94	0.50

mm9

Score	Fg/Bg	%Fg	%Bg
36	4.3	0.89	0.42

Score	Fg/Bg	%Fg	%Bg
36	4.3	0.93	0.50

Score	Fg/Bg	%Fg	%Bg
35	4.1	0.92	0.48

Score	Fg/Bg	%Fg	%Bg
35	3.4	0.91	0.51

dm3

Score	Fg/Bg	%Fg	%Bg
11.9	2.5	0.93	0.70

Score	Fg/Bg	%Fg	%Bg
8.8	1.4	1.00	0.99

Score	Fg/Bg	%Fg	%Bg
8.3	2.0	0.78	0.57

Score	Fg/Bg	%Fg	%Bg
6.9	1.6	0.81	0.63

ce10

Score	Fg/Bg	%Fg	%Bg
6.5	1.5	0.82	0.66

Score	Fg/Bg	%Fg	%Bg
6.4	1.6	0.78	0.64

Score	Fg/Bg	%Fg	%Bg
5.7	2.1	0.41	0.21

Figure S3. **A)** Top 10 features selected from the predictive model of "hotness" of genomic regions using a penalized multivariate regression method for *H.sapiens, M.musculus,*

D.melanogaster, and C.elegans. **B)** Discriminative motif discovery results between HOT and non-HOT regions in *H.sapiens, M.musculus, D.melanogaster, and C.elegans*. MotifRG was used to find longer sequence patterns that could discriminate between HOT and COLD regions. Motif discovery resulted in short, mostly G(CG) rich motifs in all four organisms. Top four most enriched motifs are shown for each organism, except C.elegans, for which there were three statistically significant motifs. Table under each motif contains the enrichment statistics for the corresponding motif: Score - motifRG calculated score, Fg/Bg, log2 ratio of percentage of foreground sequences (HOT regions) that contained the motif Vs the percentage of background sequences(non-HOT regions) containing the motif; %Fg - percentage of foreground sequences containing the motif; %Bg - percentage of background sequences containing the motif.

Figure S4

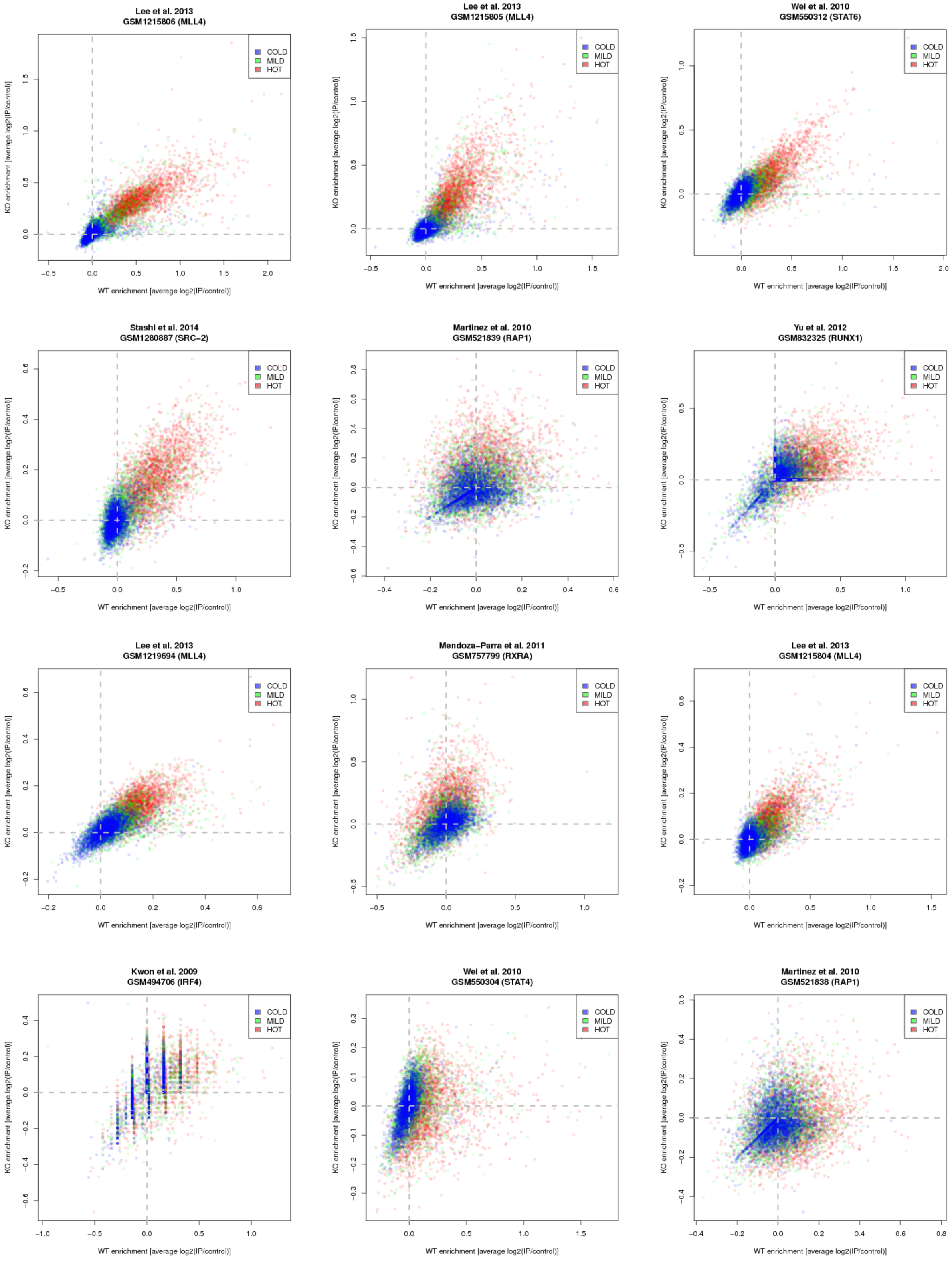
Lee et al. 2013
GSM1215806 (MLL4)
Lee et al. 2013
GSM1215805 (MLL4)
Wei et al. 2010
GSM550312 (STAT6)
Stashi et al. 2014
GSM1280887 (SRC−2)
Martinez et al. 2010
GSM521839 (RAP1)
Yu et al. 2012
GSM832325 (RUNX1)
Lee et al. 2013
GSM1219694 (MLL4)
Mendoza−Parra et al. 2011
GSM757799 (RXRA)
Lee et al. 2013
GSM1215804 (MLL4)
Kwon et al. 2009
GSM494706 (IRF4)
Wei et al. 2010
GSM550304 (STAT4)
Martinez et al. 2010
GSM521838 (RAP1)
COLD
MILD
HOT
KO enrichment [average log2(IP/control)]
WT enrichment [average log2(IP/control)]

Figure S4. Most of wild-type (WT) and knock-out (KO) ChIP-seq samples have scores that show strong correlation on HOT regions. Scatterplots show the relationship between the ChIP enrichment in WT and KO experiments for KO samples shown in Figure 3A (besides TF E2A for which we haven't found a WT experiment). Signal strength is measured as log2 RPKM ChIP / Input. The color on figures designates HOT (red), MILD (green), and COLD (blue) regions. Each dot represents one transcription factor WT peak.

Figure S5

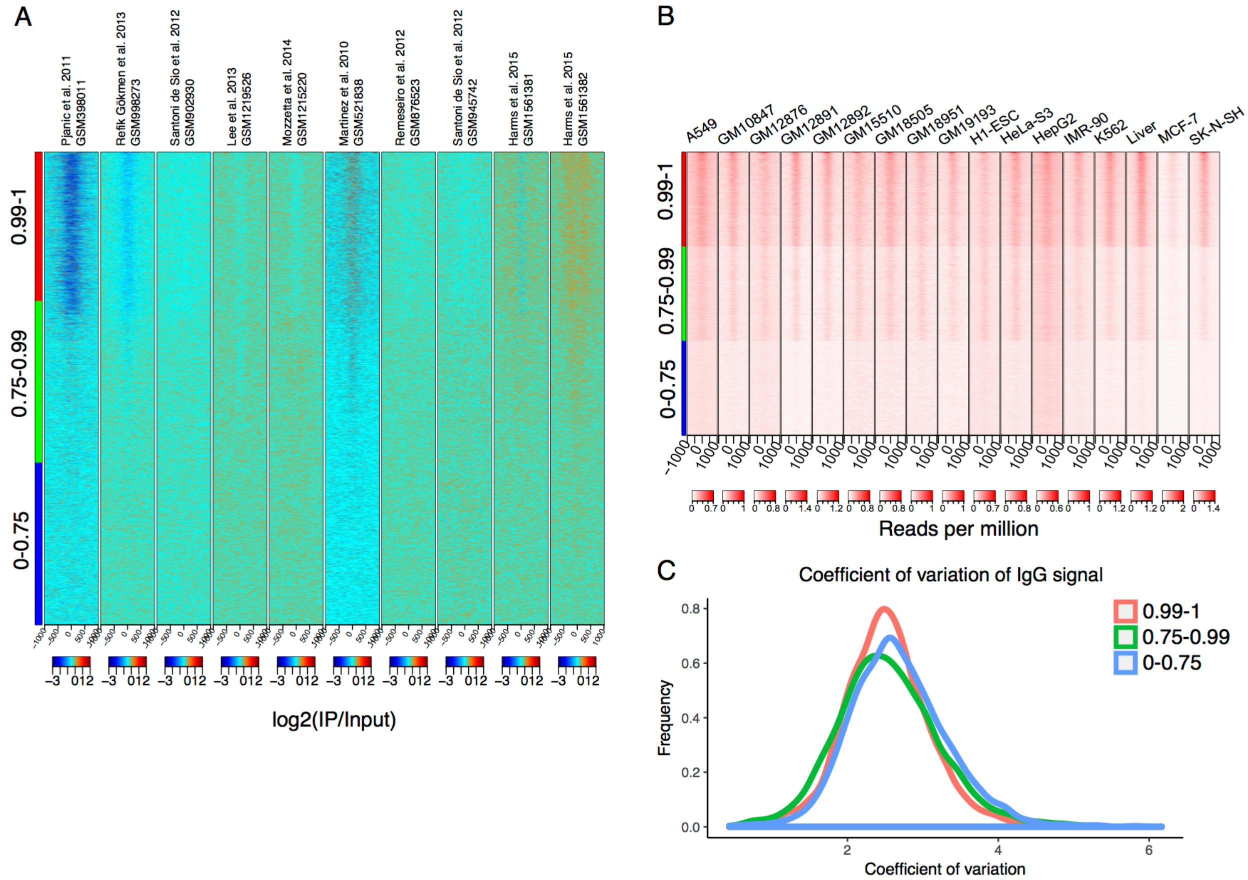

Figure S5. A) Signal profile of KO experiments where the log2 IP/Input was less than 0, around regions with decreasing transcription factor density. Specific antibodies have low signal over hot regions. **B)** Signal profile (measured as reads per million) of the IgG control antibody (ENCAB000AOJ) in multiple cell lines around regions with decreasing transcription factor density. IgG controls show an increased signal on HOT regions (0.99-1), when compared to MILD (0.75-0.99) or COLD (0-0.75), however, the signal intensity is weak and cell type dependent. **C)** Distribution of the coefficient of variation of the IgG signal for each region in B). Each color represents regions of decreasing transcription factor occupancy density (red -HOT, green - MILD, blue - COLD). If IgG showed a consistently high signal in over HOT regions in multiple cell types, the distribution of the coefficient of variation should have a right skew, when compared to the distributions over MILD and COLD regions. This is however not the case - HOT regions show the same amount of variation as do MILD and COLD regions.

Figure S6

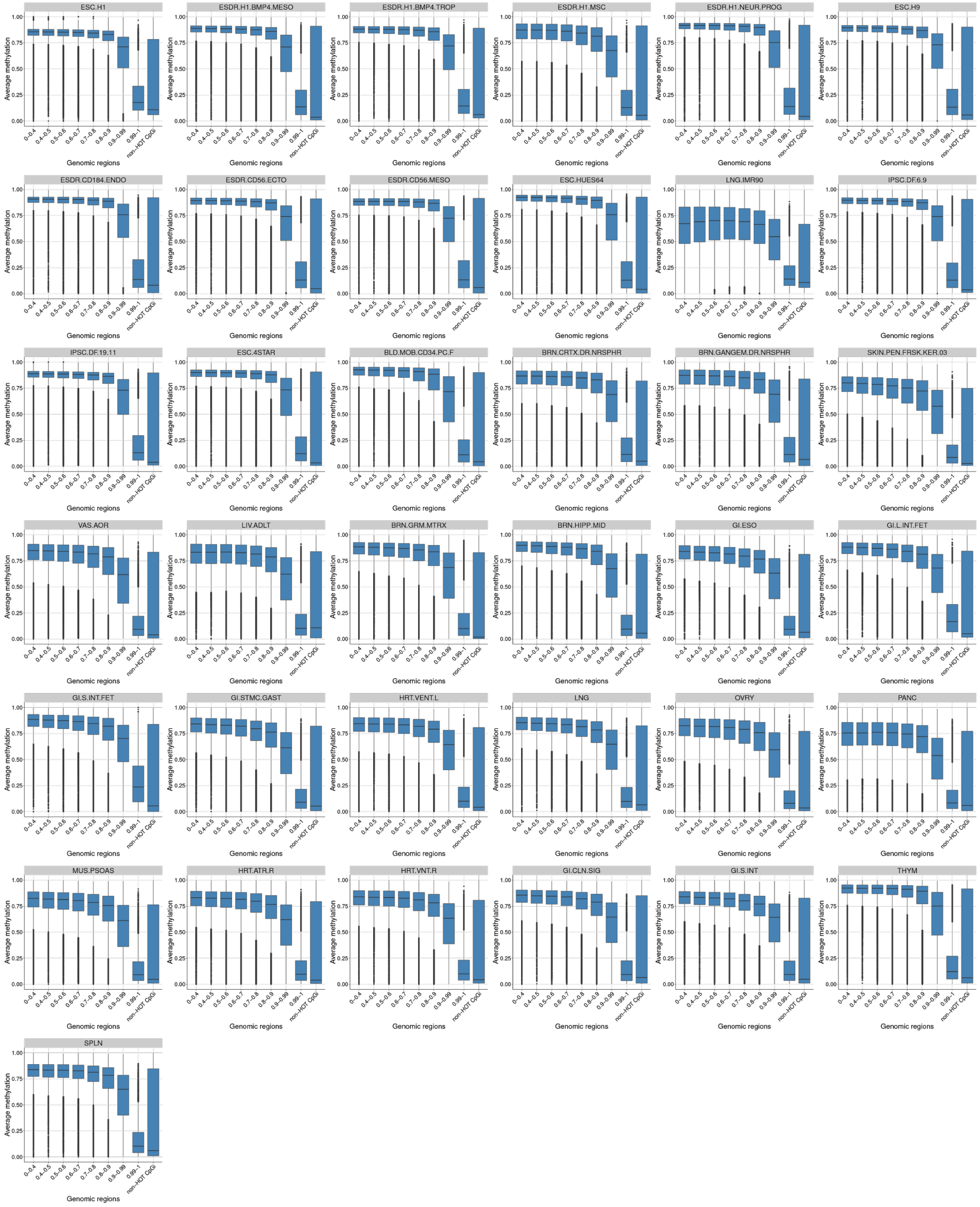

Figure S6. Average methylation level on genomic regions (HOT regions, CpG islands that are not associated with HOT regions and other genomic regions with lower TF occupancy) for 37 human cell lines derived from the Roadmap Epigenomics database.
